# FERONIA homologs in stress responses of horticultural plants: current knowledge and missing links

**DOI:** 10.1007/s44154-024-00161-1

**Published:** 2024-06-07

**Authors:** Xinhua Huang, Yuhan Liu, Yanhong Jia, Lizhu Ji, Xiaomin Luo, Shiping Tian, Tong Chen

**Affiliations:** 1 State Key Laboratory of Plant Diversity and Specialty Crops, Institute of Botany, Chinese Academy of Sciences, Beijing, 100093 China; 2 China National Botanical Garden, Beijing, 100093 China; 3 University of Chinese Academy of Sciences, Beijing, 100049 China; 4 Vegetable Research Institute, Tianjin Academy of Agricultural Sciences, Tianjin, 300384 China

**Keywords:** Receptor-like kinase, FERONIA, Stress responses, Horticultural crops

## Abstract

Owing to its versatile roles in almost all aspects of plants, FERONIA (FER), a receptor-like kinase of the *Catharanthus roseus* receptor-like kinase 1-like (*Cr*RLK1L) subfamily, has received extensive research interests during the past decades. Accumulating evidence has been emerged that FER homologs in horticultural crops also play crucial roles in reproductive biology and responses to environmental stimuli (abiotic and biotic stress factors). Here, we provide a review for the latest advances in the studies on FER homologs in modulating stress responses in horticultural crops, and further analyze the underlying mechanisms maintained by FER. Moreover, we also envisage the missing links in current work and provide a perspective for future studies on this star protein.

## Introduction

Plants have gradually evolved a complete set of sophisticated signaling mechanisms to cope with complex environment cues, during which receptor-like kinases (RLKs) have great contributions. RLKs are a group of surface-localized, transmembrane receptors comprising large families of well-studied kinases. They exert their functions through transmembrane and juxtamembrane domains with the aid of various interacting partners and downstream components (Zhou and Zhang 2020). In recent years, FERONIA (FER), a member of the *Catharanthus roseus* receptor-like kinase 1-like (*Cr*RLK1L) protein kinase subfamily, has appealed extensive research interests (Wang et al. 2022; Ma et al. 2023). This protein regulates sexual reproduction process, growth and development, responses to abiotic and biotic stress, etc., which has been well known as a star protein in plant biology (Zhu et al. 2021).

Horticultural crops, including fruits, vegetables, tea and ornamental plants, provide abundant nutrients, dietary fibers and ornamental values, thereby benefiting our daily life quality (Xu et al., 2022). However, adverse environmental factors dramatically affect the production of horticultural products, resulting in quality deterioration and ultimately economic losses. Recent studies have shown that FER homologs also play versatile roles in various aspects in horticultural crops, regulating sexual reproduction processes, normal development, responses to biotic and abiotic factors (Zhang et al. 2021; Ji et al. 2023; Jing et al. 2023a, b). This review mainly focuses on the advances in the studies on FER homologs among horticultural crops, focusing on the function and molecular mechanism of FER homologs in regulating the interaction between horticultural plants and stress factors. These results may deepen our understanding towards the mechanism of RLKs in regulating the stress responses and provide references for molecular improvement of horticultural crop quality.

## FERONIA homologs in horticultural crops and their phylogenetic relationship

As one of its sequence characteristics, FER has no intron, which has been extensively found in eukaryotic cells to avoid of R-loop formation and maintain DNA stability during transcription (Niu 2007; Gozashti et al. 2022). Alternatively, the absence of intron in FER homologs also implies that these homologs may be highly conserved in sequences and possibly their functions as compared with those in model plants. Almost all FER homologs consist of a signal peptide, two malectin-like domains in their extracellular domain, a transmembrane domain and an intracellular kinase domain, which strongly indicate their functions in phosphorylation events as RLKs.

## Functions of FERONIA homologs in horticultural crops

### Roles of FER homologs in growth and development of horticultural crops

**Fig. 1:**
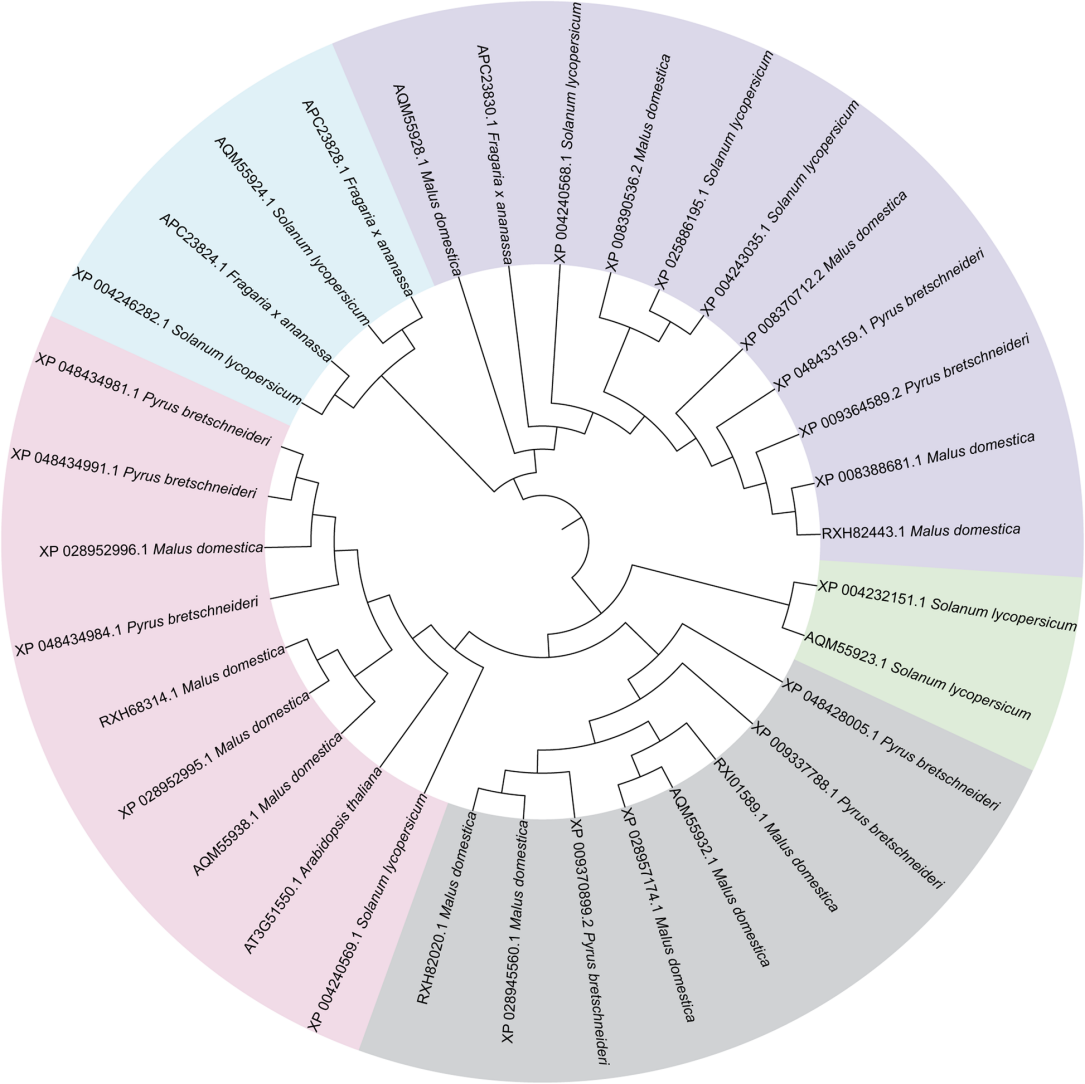
Phylogenetic analysis of FER homologs in *S. lycopersicum*, *M. dosmetica*, *F.* × *ananassa* and *P. bretchneideri*. The sequences were downloaded from National Center for Biotechnology Information (NCBI, https://www.ncbi.nlm.nih.gov/) (Bethesda, MD, USA, accessed on Feb 2, 2024) and Sol Genomics Network (SGN, https://solgenomics.net/) (Ithaca, NY, USA, accessed on Feb 2, 2024)

As shown in Fig. 1, several recently reported FER homologs are clustered in the same clade with AtFER, suggesting that they may share similar functions. Originally reported as a regulator of double fertilization in Arabidopsis, FER has important roles in the rejection of self-pollen and the acceptance of compatible-pollen. An excellent study revealed that stigmatic reactive oxygen species (ROS) level got burst after self-pollination but decreased after compatible-pollination in Chinese cabbage (*Brassica rapa* L. ssp. *pekinensis*). These ROS-mediated responses were controlled by FER1-Rop2 module in a NADPH oxidase (RbohF) dependent manner, suggesting a relatively conserved role of FER-Rac/Rop GTPase-NADPH oxidase in regulating self-incompatibility in Cruciferous plants (Zhang et al. 2021).

FER homologs have important roles in fruit development and ripening. Following homology-based analysis, Jia et al. identified MdFERL6 and MdFERL1, two homologs of Arabidopsis FER, interacted with MdSAMS (S-adenosylmethionine synthase), an enzyme catalyzing the first step in ethylene biosynthesis. MdFERL6 was expressed highly during early fruit development, but dramatically declined when fruit ripening commenced, implying that MdFERL6 might limit ethylene production prior to fruit development and further induce ethylene production burst during fruit ripening (Jia et al. 2017a). However, the mechanism underlying such variation is still unclear. As a typical non-climacteric fruit, *Fragaria* × *ananassa* is highly favored by consumers worldwide. FaMRLK47, a FER-like receptor kinase, was found to regulate quality formation in strawberry fruit ripening. FaMRLK47 interacted with FaABI1, a negative regulator of abscisic acid (ABA) signaling, thereby affecting fruit ripening by modulating ABA signaling (Jia et al. 2017b). Similarly, by combining Surface Plasmon Resonance (SPR) assay and mass spectrometry, Ji et al. reported that the transcriptional complex RIN-TAGL1 activated *SlFERL* upon the onset of fruit ripening. SlFERL recruited SlSAMS1 to the plasma membrane and further promoted ethylene biosynthesis during tomato fruit ripening (Ji et al. 2020a, b). However, they did not identity whether SlFERL may phosphorylate SlSAMS1, although four of the five predicted phosphorylated sites were detected free of phosphorylation modification following interaction. In addition, different from the variation patterns for *MdFERL1* and *MdFERL6* during fruit ripening, *SlFERL* was persistently upregulated, suggesting possibly different regulatory machinery. Alternatively, this may be also attributed to potentially redundant functions of homologs in apple.

### FER homologs regulate host–pathogen interaction

**Fig. 2:**
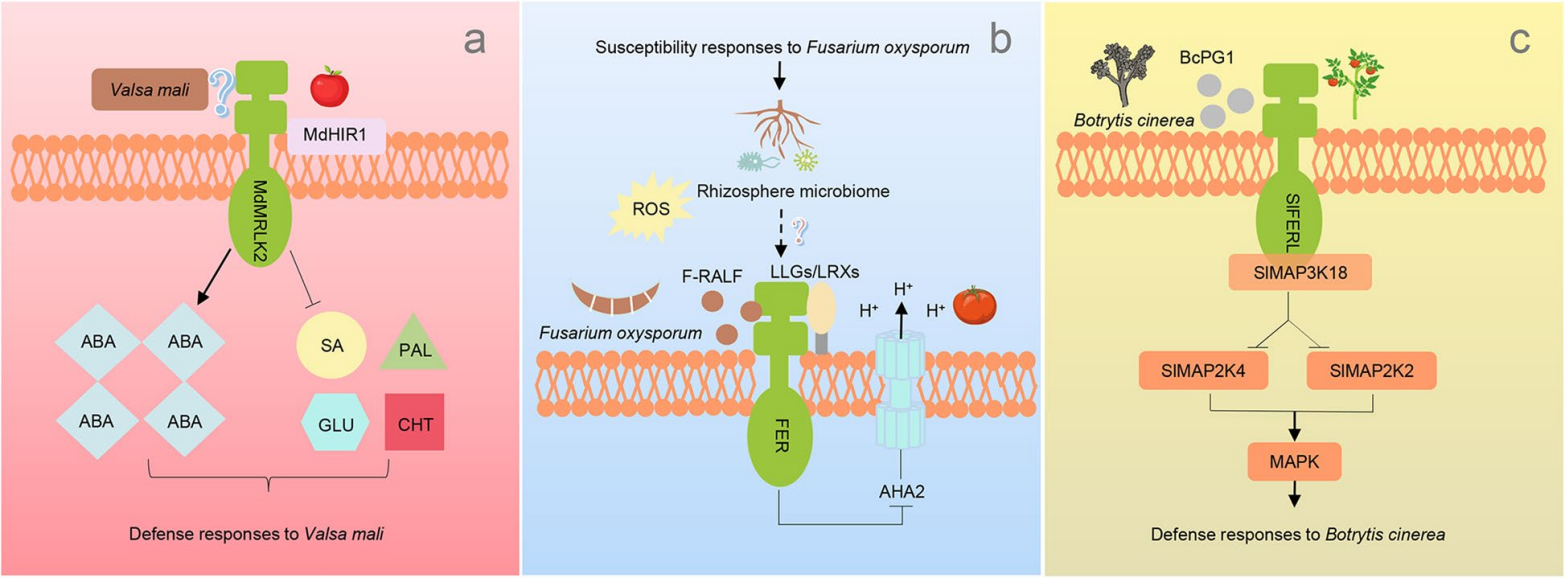
FER homologs are extensively involved in the interaction with pathogens of different life styles. **a** MdMRLK2 interacts with MdHIR1, thereby modulating ABA and SA levels, phenolic contents as well as a bulk of degrading enzymes to affect the response to *V. mali*; **b** FER recognizes F-RALF secreted by *F. oxysporum* and further induces extracellular alkalinization by suppressing AHA2 activity, which ultimately promotes the enrichment of certain rhizosphere microbiota as well as growth and resistance of host plants, while further evidence is still required to ascertain whether LLG/LRXs also function in this context, as shown in model plants; **c** SlFERL interacts with BcPG1, a virulence factor secreted by *B. cinerea*, to fine tune MAPK signaling cascade by recruiting SlMAP3K18 and fine-tuning MAP2K protein level and activity in response to *B. cinerea* invasion

Aside from the roles in developmental processes, FER homologs are widely involved in the interaction between pathogens and host plants, thus being regarded as a “busy goodness” (Fig. 2). To facilitate their colonization, pathogens always employ versatile strategies to accomplish infection, depending on their trophic types. As early as 2010, evidence was reported that the knockout mutant of *AtFER* displayed unchanged susceptibility to *Hyaloperonospora arabidopsidis* and *Colletotrichum higginsianum*, but higher resistance to *Golovinomyces orontii*, a typical biotrophic pathogen (Kessler et al. 2010). Although the mechanism has been partly attributed to the elevated ROS level and the spontaneous cell death, the components involved in this resistance are still enigmatic until now. A recent study showed that phospholipase D (PLD) δ-derived phosphatidic acid (PA) contribute to the regulation on the foci of certain secretory proteins at the site of penetration (Xing et al. 2019). It wound be interesting to investigate whether FER homologs may also have endocytic activity in response to powdery mildew infection, as some results have been documented that FER recycled between plasma membrane (PM) and intracellular structures in response to flg22 stimuli (Xing et al. 2022).

Meanwhile, a bulk of data have been reported on the functions of FER homologs in regulating the interaction between hemi-biotrophic pathogens and host plants. *Fusarium oxysporum*, a hemi-biotrophic root-infecting fungus, uses Fusarium (F)-RALF (rapid alkalinization factor) peptide to hijack host FER and induce extracellular alkalinization by blocking the PM-localized H^+^-ATPase 2 (AHA2) activity (Haruta et al. 2014). The resulted alkalinization facilitated Fusarium infection by stimulating phosphorylation of Fmk1, a conserved mitogen-activated protein kinase (MAPK) essential for pathogenicity. Consequently, F-RALF caused growth arrest of roots, whereas *fer-4*, a knockout mutant lacking a functional FER, displayed enhanced resistance against Fusarium (Masachis et al. 2016). Interestingly, RALF-like peptides have been reported in many phylogenetically distant groups of fungi, or even root-knot nematode. *Meloidogyne incognita* secretes two RALF-likes peptides (i.e., MiRALF1 and MiRALF3) (Zhang et al. 2020). These small peptides can also bind to the extracellular domain of FER, thereby modulating downstream responses and cell expansion required for further parasitism (Zhang et al. 2020), which partly explains for the lower susceptibility of *fer-4* mutant s to *M. incognita*. All these results implied that different RALFs or RALF-like peptides may possess conserved N-terminal motifs that are recognized in a similar manner, as revealed by Xiao et al., that the N terminus of RALF23 has higher affinities for LORELEI (LRE)-LIKE GLYCOSYLPHOSPHATIDYLINOSITOL (GPI)-ANCHORED PROTEIN (LLG)1–3 and FER, functioning as an “adhesive” to facilitate the formation of complex between LLGs and FER (Xiao et al. 2019; Ge et al. 2019; Zhang et al. 2023).

In addition, *C. acutatum* is another hemi-biotrophic pathogen causing anthracnose on strawberry. FaRALF33-like expression was up-regulated in ripe fruit at 24 h upon *C. acutatum* inoculation, whereas no difference was observed following *P. expansum* inoculation. Moreover, the *C. acutatum*-inoculated ripe fruit showed higher FaRALF33-like level as compared to the mock fruit. In contrast, the expression level of *C. acutatum*-inoculated ripe fruit at 48 h was resemble to the mock fruit (Merino et al. 2019). However, there is no direct evidence to indicate whether FaRALF33-like may bind to FER homologs to trigger these responses, which deserves further in-depth dissection. When *C. acutatum* could not secrete ammonia at early infection, it may induce RALF expression in susceptible ripe fruit to facilitate its pathogenicity. The authors hypothesized that fungi may also co-opt the host RALF-mediated pathway, instead of coding of RALF homologs in their own genomes (Masachis et al. 2016). The susceptibility modulated by RALF against *C. acutatum* in ripe fruit may be explained by the altered ambient pH following the quiescence state.

Noteworthy, Song et al. found that *hsm*13 mutant (also known as *fer-8*) showed dwarfness and rhizosphere enrichment of *Pseudomonas fluorescens* WCS365. The microbiome transplant assay showed that the microbiome of *fer-8* was beneficial and independent of the previously reported scaffolding function and jasmonic acid (JA)-mediated immunity. Moreover, the roots of *fer-8* mutant had lower levels of ROS, which may be responsible for the enrichment of *Pseudomonads*, while the supplement with RALF23 restored the phenotype of *P. fluorescens* enrichment (Song et al. 2021). These results further broaden our understanding towards the ambient pH and microbiome signaling modulated by FER. Alternatively, in attempt to identify the potential interacting proteins of LeEIX2 (ethylene-inducing xylanase), SlRLK-like, also a *FER* homologous gene in the *Cr*RLK1 family, was retrieved as a target (Sussholz et al. 2020). The responses induced by EIX were markedly reduced in the SlRLK-like overexpressing plants, while the knockout lines showed increased EIX-induced ethylene production and 1-aminocyclopropane-1-carboxylate synthase (SlACS2) expression. Additionally, co-expression of SlRLK-like with LeEIX2, FLS2, Ve1 and AtRLP23 led to a reduction in the abundance of these pattern recognition receptors and further attenuation of pattern-triggered immunity, thereby implying a potential role of SlRLK-like in signaling desensitization. However, the underlying mechanism remains largely unresolved and some potentially key players as narrated in model plants in this process are still unidentified.

Necrotrophic pathogens are among the most notorious enemies for horticultural crops. Apple trees are highly susceptible to *Valsa mali*, a typical necrotrophic pathogen. As reported, *MdMRLK2*, a *FER* homologous gene, was induced by *V. mali* in the susceptible cultivar as compared with the resistant cultivar (Jing et al. 2022). The *MdMRLK2*-overexpressing plants exhibited lower resistance as compared to wild-type plants, which was further attributed to the higher abscisic acid (ABA) and lower salicylic acid (SA) levels. Meanwhile, some specific phenolic substances, phenylalanine ammonia-lyase, β-1,3-glucanase, and chitinase activities also varied dramatically upon *V. mali* infection, which may be solely dependent on MRLK2 function. Moreover, MdMRLK2 interacted with MdHIR1, a hypersensitive-induced response protein resided in the so-termed membrane nanodomain (Keinath et al. 2010; Li et al. 2012), ultimately suppressing the hypersensitive response (HR) mediated by MdHIR1. These findings collectively pointed out that FER homologs function with specific components in immune complexes to fine-tune their stability. *B. cinerea* is another necrotrophic pathogen causing severe pre- and post-harvest gray mold on many important horticultural crops (Gamir et al., 2021; Chen et al. 2023b). Although the *fer-4* mutant showed higher cuticle permeability and increased resistance to *B. cinerea* (Lorrai et al., 2021), another line of evidence has also been demonstrated that unchallenged *Atfer* and *Slferl* leaves displayed spontaneous cell death and H_2_O_2_ burst (Kessler et al. 2010; Ji et al. 2023). Ji et al., reported that the extracellular domain of SlFERL perceived BcPG1, a virulence protein secreted by *B. cinerea*, further phosphorylated SlMAP3K18 to trigger MAPK cascade (Ji et al. 2023). Notably, BcPG1 triggered defense response of tomato, which was dependent on SlFERL but independent of the pectin-hydrolyzing activity of BcPG1. Alternatively, the authors also noted that SlFERL may be hijacked by *B. cinerea* for its function to induce cell death, which largely depended on the activated MAPK signaling. This further complicates the infection strategies of *B. cinerea* as a representative of necrotrophic pathogen. Recently, a study depicted that RALF22 triggered immune responses and augmented Pep3-induced signaling in a FER-dependent manner in Arabidopsis (He et al. 2023), suggesting that RALF22 may be utilized as an elicitor to *Sclerotinia sclerotiorum*, also a devastating necrotrophic pathogen.

### FER homologs modulate responses to abiotic stress

**Fig. 3:**
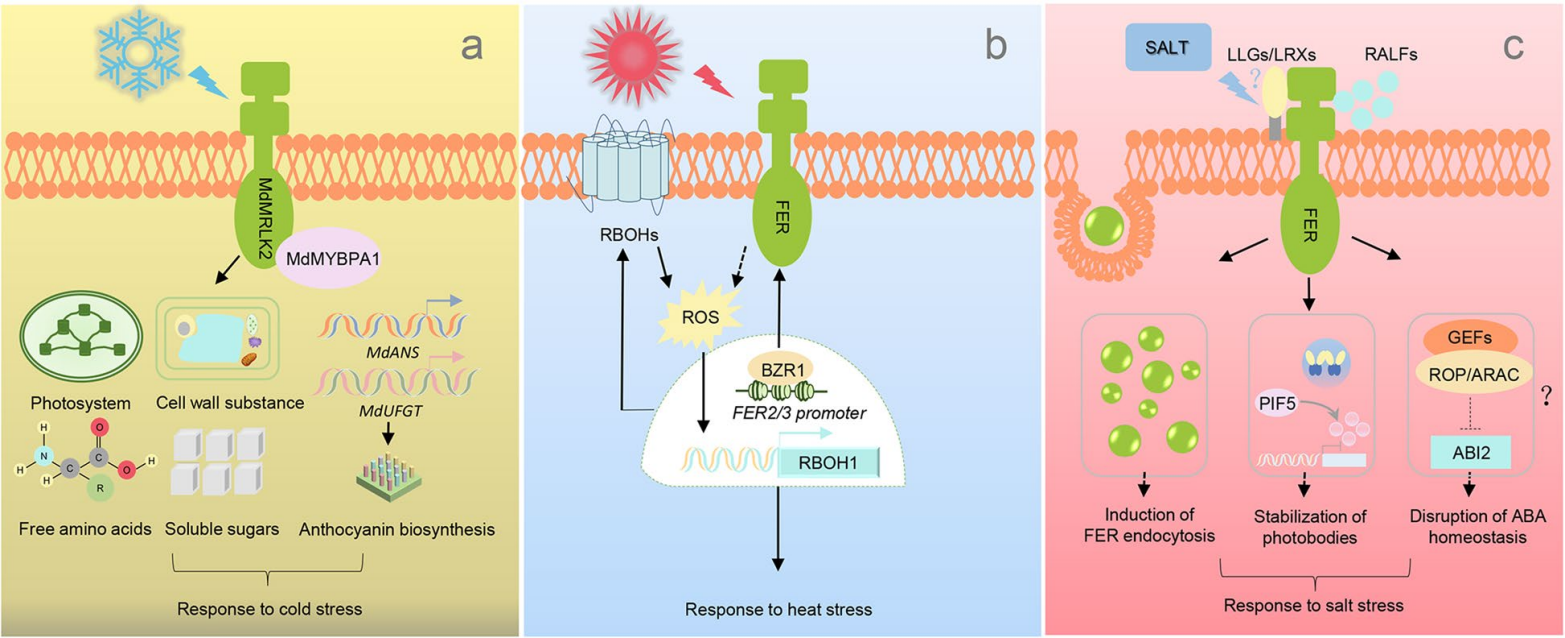
FER homologs modulate responses to cold, high temperature and salinity stress. **a** *MdMRLK2* overexpression line shows higher tolerance to cold stress, which was attributed to the interaction with MdMYBPA1 and further modulation of anthocyanin biosynthesis, contents of cell wall components, soluble sugars and amino acids as well as photosystem efficiency; **b** BZR1 transcriptionally activates *FERONIA2* (*FER2*) and *FER3* to modulate RBOH1-dependent ROS production and responses to high temperature; **c** *MdFER* overexpression improves the tolerance of apple calli to salinity stress and reduced the sensitivity to ABA via a currently unidentified mechanism, although evidence has been provided that FER mediates the interaction between ROPs/ARAC and ABI2 as well as stabilization of photobodies, or undergoes endocytosis upon exposure to NaCl, thus being involved in salt stress responses in Arabidopsis. Moreover, it is still unclear whether a similar mechanism involving LLGs/LRXs may exist in horticultural crops upon exposure to salinity stress

Plants are sessile in soil during their whole lifetime, obliging them to evolve sophisticated signal perception and transduction networks to deal with changing environmental conditions. Results have been shown that FER is pleiotropic in its functions in model plants in response to cold, heat and salt stress (Chen et al. 2016; Feng et al. 2018; Richter et al. 2017; Mustamin et al. 2023). It has been recently found that FER homologs also modulate responses to abiotic stress, e.g., FER mediates the interaction between ROP11/ARAC10 and ABI2, thereby affecting auxin and ABA signaling cross-talk (Yu et al. 2012). Moreover, FER also interacts with several guanine nucleotide exchange factors (GEFs) and ROPGEF1 further interacts with RAC/ROP to modulate root hair development (Duan et al. 2010). Although the modulation of FER-RAC/ROP module has also been reported in *B. rapa* (Zhang et al. 2021), it is still unknown up to now whether this module may work as a common mechanism (Fig. 3).

In response to cold stress, *MdMRLK2* was rapidly induced and the apple plants overexpressing *MdMRLK2* displayed higher tolerance. This was attributed to higher contents of major wall components, including cellulose, hemicellulose, pectins and lignin. Meanwhile, higher soluble sugars and amino acids and less damage to photosystem were also observed in the 35S:*MdMRLK2* overexpressing plants. Moreover, MdMRLK2 interacted with the transcription factor MdMYBPA1 to promote the activation of *MdANS* and *MdUFGT*, leading to higher anthocyanin production, especially upon cold stress (Jing et al. 2023a, b). These results imply a possibility that FER homologs or truncates may migrate into the nucleus and interact with potential interactors under specific scenarios. However, this hypothesis still requires further experimental supports. *MdMRLK2* was also significantly upregulated by ABA and drought stress. 35S:*MdMRLK2* lines exhibited higher photosynthetic rates and enhanced energy levels under drought conditions, which was resulted from activated caspase activity, higher levels of free amino acids and higher ABA content (Jing et al. 2023a, b). These findings further confirm that FER homologs are pleiotropic to respond to various environmental stimuli. Moreover, being a single-copy gene with coding region SNPs in *Lotus japonicus*, *LjFER* showed haplotype-dependent cold-responsive expression (Mustamin et al. 2022).

High temperature often leads to yield loss and quality deterioration in crops. It has been previously reported that HERCULES1, THESEUS1, and FER, three RLKs in the *Cr*RLK family, are transcriptionally induced by brassinosteriods (BRs) and are down-regulated in *bri1*, suggesting certain correlations between these RLKs with the BR signaling pathway (Guo et al. 2009). BRASSINAZOLE RESISTANT 1 (BZR1), a key regulator of BR signaling, could bind to the promoters of *FERONIA2* (*FER2*) and *FER3* and activated their expression, while it has been documented that FER functions in the upstream of ROP signaling to modulate NADPH oxidase and ROS burst (Duan et al. 2010; Xia et al. 2015). Silencing of *FER2/3* suppressed the induction of *RBOH1* transcripts, extracellular H_2_O_2_ accumulation and heat tolerance (Yin et al. 2018). However, it still requires further evidence to ascertain how *FER2* and *FER3* are involved in extracellular H_2_O_2_ production upon exposure to high temperature.

Salinity stress is another unfavorable environmental factor seriously affecting the yield of horticultural crops. FER homologs are also involved in the signaling in response to salt stress. The expression of *MdFER*, a *FER* homologous gene predominantly expressed in roots, was significantly induced by NaCl and ABA. Moreover, *MdFER* overexpression dramatically improved the salt tolerance of calli and reduced the sensitivity to ABA (Xie et al. 2022). However, interactors involved in the responses to salinity are still largely unknown, as it may be expected that the components functioning to induce FER endocytosis and stabilize photobodies in model species may also work in horticultural crops, and it would also be interesting to examine whether leucine-rich repeat extensins may be involved in this event (Zhao et al. 2018; Liu et al. 2023). Notably, a recent study depicted the global signaling capacity of FER mediated by a previously undescribed extracellular phase separation process driven by RALF-pectin interaction. This process encompasses receptor plasma membrane clustering events and subsequent endocytic activity, which composes a possibly common mechanism in response to salinity, drought and probably other stress conditions (Liu et al. 2024).

## Missing links in the mechanisms involving FERONIA homologs

Although the past several decades have witnessed exciting achievements in elucidating the roles of FER homologs in horticultural crops, there are still some missing links to fully understand the exact roles: a) Given its autophosphorylation feature, numerous interacting proteins and implications in various biological processes, it would be interesting to identify the global phosphorylation atlas and the involved substrates at a genome-wide or proteome-wide level, and whether the phosphorylation of protein substrates may also function as a part of the mechanisms for signaling specificity maintained by FER-substrate modules; b) Could FER homologs recognize other conserved molecular patterns from beneficial or harmful microorganisms, and other macromolecules from plants? c) Desensitization of immune signaling is essential for maintaining normal growth and development, but molecular components involved in signaling attenuation are still largely missing. Modulation of endocytic activity may be one of the possible machineries, as it has been reported that FER may undergo endocytic activity in response to flg22 stimulus and stress factors (Xing et al. 2022; Liu et al. 2024); d) Downstream targets regulated by FER homologs are still far from completion, especially for those involved in secondary metabolite biosynthesis. It should be noticed that FER may be cleaved by a metalloproteinase to release the kinase domain into the nucleus when RALF23 accumulates in transition/elongation zone (TZ/EZ) cells during bacterial invasion (Chen et al. 2023a). Although the underlying mechanism has not been fully elucidated, this finding also raises new questions for this nuclear localization of FER or its truncate.

## Summary and prospect

As illustrated above, like their counterpart in Arabidopsis and other model species, FER homologs also play versatile roles in the stress responses of horticultural crops. However, it still deserves further efforts to examine whether the mechanisms in model species also work in horticultural crops, and how various signaling events are modulated in high specificity by FER and its interacting proteins. Evidence has been shown that the contribution of FER in monitoring turgor-dependent cell wall tension as a mechanosensor may explain for its pleiotropy (Malivert and Hamant, 2023). We can anticipate that there is still a long way to ascertain the upstream signaling molecules perceived by FER and the downstream interactors as kinase substrates. Nevertheless, based on the mechanisms underlying FER homolog functions, it may be exciting to take advantage of state-of-art precise gene editing techniques to generate horticultural crops with higher quality and resistance without growth penalty. 

## Data Availability

All data generated or analyzed during this study are included in this published article.
